# Supplementary material for: Asynchronous magnetic resonance elastography: Shear wave speed reconstruction using noise correlation of incoherent waves
Source: Magn Reson Med. 2022 Oct 27;89(3):990–1001. doi: 10.1002/mrm.29502 (PMC9792433; doi:10.1002/mrm.29502)
Supplement: Supplementary file 1 — DATA S1 MATLAB code used to generate the numerical simulations of Figure 1 [file MRM-89-990-s002.zip › k-Wave/helpfiles/example_pr_2D_tr_directional_sensors.html]

Image Reconstruction With Directional Sensors Example (k-Wave)


# Image Reconstruction With Directional Sensors Example

This example demonstrates how the directionality of sensor elements can give rise to artefacts in time reversal photoacoustic image reconstruction.
It builds on the Sensor Element Directivity in 2D and 2D Time Reversal Reconstruction For A Line Sensor examples.

- Open the file in the MATLAB Editor
- Run the file in MATLAB

For a more detailed discussion of this example and the underlying techniques, see B. T. Cox and B. E. Treeby, "Effect of sensor directionality on photoacoustic imaging: A study using the k-Wave toolbox," in *Proc. SPIE*, vol. 7564, p. 75640I, 2010.

## Contents

- Running the simulation
- Time-reversal image reconstruction

## Running the simulation

The simulation is first invoked using a rectangular binary sensor mask with omnidirectional elements in the same way as previous examples. Directionality is then added to the sensor elements by assigning an angle to the corresponding elements in an Nx by Ny matrix passed to `sensor.directivity_angle`. These are assigned such that the maximum sensitivity of each sensor face faces inward.

```
% define a four-sided, square sensor
sensor.mask = zeros(kgrid.Nx, kgrid.Ny);
sensor.mask(1, :) = 1;
sensor.mask(end, :) = 1;
sensor.mask(:, 1) = 1;
sensor.mask(:, end) = 1;

% run the simulation for omnidirectional detector elements
sensor_data = kspaceFirstOrder2D(kgrid, medium, source, sensor, input_args{:});

% define the directionality of the sensor elements
sensor.directivity_angle = zeros(kgrid.Nx, kgrid.Ny);
sensor.directivity_angle(1, :) = 0;      % max sensitivity in x direction
sensor.directivity_angle(end, :) = 0;    % max sensitivity in x direction
sensor.directivity_angle(:, 1) = pi/2;   % max sensitivity in y direction
sensor.directivity_angle(:, end) = pi/2; % max sensitivity in y direction

% define the directivity size
sensor.directivity_size = 20 * kgrid.dx;

% run the simulation with directional elements
sensor_data_directional = kspaceFirstOrder2D(kgrid, medium, source, sensor, input_args{:});
```

## Time-reversal image reconstruction

To examine the effect of sensor directionality on time reversal image reconstruction, the sensor data recorded from both the omni-directional and directional sensor elements is used to reconstruct the initial photoacoustic pressure distribution. The reconstructed pressure distributions are shown below. For the omni-directional sensor, the initial pressure is reconstructed almost exactly (note, the inverse crime in which the same simulation parameters are used in both simulation and reconstruction has been committed). However, when using the directional sensor, image artefacts are introduced into the reconstruction.
